# Personalizing Neoadjuvant Chemotherapy: The Impact of BRCA Variants on Pathologic Complete Response in Luminal B Breast Cancer

**DOI:** 10.3390/cancers17101619

**Published:** 2025-05-10

**Authors:** Alba Di Leone, Antonio Franco, Virginia Castagnetta, Marta Silenzi, Cristina Accetta, Beatrice Carnassale, Sabatino D’Archi, Flavia De Lauretis, Enrico Di Guglielmo, Federica Gagliardi, Stefano Magno, Francesca Moschella, Maria Natale, Alejandro Martin Sanchez, Lorenzo Scardina, Riccardo Masetti, Gianluca Franceschini

**Affiliations:** Multidisciplinary Breast Center, Department of Women, Children and Public Health Sciences, Fondazione Policlinico Universitario Agostino Gemelli IRCCS, Università Cattolica del Sacro Cuore, 00168 Rome, Italy; alba.dileone@policlinicogemelli.it (A.D.L.); antonio.franco@guest.policlinicogemelli.it (A.F.); martasilenzi@tiscali.it (M.S.); cristina.accetta@policlinicogemelli.it (C.A.); beatrice.carnassale@guest.policlinicogemelli.it (B.C.); sabatino.darchi@policlinicogemelli.it (S.D.); flavia.delauretis@gmail.com (F.D.L.); enricodiguglielmo@yahoo.com (E.D.G.); federica.gagliardi@uniroma1.it (F.G.); stefano.magno@policlinicogemelli.it (S.M.); francesca.moschella@policlinicogemelli.it (F.M.); maria.natale@policlinicogemelli.it (M.N.); martin.sanchez@policlinicogemelli.it (A.M.S.); lorenzo.scardina@policlinicogemelli.it (L.S.); riccardo.masetti@policlinicogemelli.it (R.M.); gianlucafranceschini70@gmail.com (G.F.)

**Keywords:** BRCA, luminal B, neoadjuvant chemotherapy, pathologic complete response

## Abstract

**Background**: Neoadjuvant chemotherapy (NACT) is effective in downstaging locally advanced breast cancer, improving surgical and oncological outcomes. However, luminal B breast cancer typically exhibits a poorer response to NACT, with only 10–15% of patients achieving a pathologic complete response (pCR). This study investigates whether BRCA pathogenic variants (BRCA PVs) influence pCR rates in luminal B breast cancer patients, aiming to identify potential predictors for personalized treatment strategies. **Materials and Methods**: This retrospective study included luminal B breast cancer patients who underwent NACT at the Fondazione Policlinico Universitario Agostino Gemelli IRCCS between January 2014 and June 2023. Patients were stratified according to BRCA status: BRCA PVs and BRCA wild-type (WT). Primary endpoint was to evaluate pCR rates, while secondary endpoints included locoregional disease-free survival (LR-DFS), distant disease-free survival (DDFS), and overall survival (OS). **Results**: In total, 495 patients were enrolled, of whom 442 (89.3%) carried BRCA WT and 53 (10.7%) BRCA PVs. The pCR rate was significantly higher in the BRCA PVs group (20.8% PVs vs. 10.9% WT; *p* = 0.044). Specifically, the breast pCR rate was 28.3% in BRCA PVs versus 15.4% in BRCA WT (*p* = 0.030). BRCA WT patients had better 5-year LR-DFS (91.1% WT vs. 79.5% PVs; *p* = 0.003), while no significant differences were observed in 5-year DDFS or OS. **Conclusions**: BRCA PVs are associated with a higher pCR rate in luminal B breast cancer patients receiving NACT, suggesting a potential predictive role in tailoring treatment strategies.

## Simple Summary

Breast cancer treatment is not the same for everyone, and understanding who benefits most from specific therapies is crucial. Neoadjuvant chemotherapy, given before surgery, can shrink tumors and improve surgical options, but its effectiveness is variable. In hormone receptor-positive HER2-negative breast cancer, one of the most common subtypes, patients with BRCA gene mutations may respond differently to treatment. This study analyzed nearly 500 patients to investigate whether BRCA mutations influence response to neoadjuvant chemotherapy. The results revealed that patients with BRCA mutations were twice as likely to experience pathologic complete response compared to those without mutations. These findings suggest that BRCA status could help personalize treatment decisions, ensuring that the right patients receive the most effective therapy.

## 1. Introduction

Breast cancer remains a leading cause of cancer-related mortality among women globally, characterized by diverse molecular subtypes that exhibit distinct biological behaviors and responses to treatment. Among these, luminal B breast cancer is particularly notable, defined by hormone receptor (HR) positivity, human epidermal growth factor receptor 2 (HER2) negativity, and elevated proliferation rates. This subtype accounts for approximately 60% of all breast cancers [1] and poses significant challenges in clinical management due to its aggressive nature [2].

Neoadjuvant chemotherapy (NACT) has emerged as a standard approach for downstaging locally advanced breast cancer, improving surgical outcomes, and providing effective systemic therapy. Additionally, compared to the adjuvant approach, NACT offers the advantages of real-time monitoring and confirmation of treatment effect in terms of pathologic complete response (pCR) [3]. However, patients with luminal B breast cancer often demonstrate suboptimal responses to NACT, with only 10–15% achieving a pathologic complete response (pCR) [4]. This limited pCR rate is concerning as pCR is strongly correlated with favorable long-term outcomes, including enhanced event-free survival and overall survival [5]. Whether NACT offers superior outcomes to adjuvant chemotherapy for these patients continues to be debated.

A critical factor influencing treatment response in breast cancer is the presence of pathogenic variants (PVs) in the BRCA genes (BRCA1 and BRCA2) [6]. These variants disrupt DNA repair mechanisms, rendering tumors more vulnerable to DNA-damaging agents used in chemotherapy regimens. Recently, studies have indicated a significant association between BRCA PVs and increased rates of pCR in luminal B breast cancer patients undergoing NACT, suggesting that these genetic alterations could serve as potential biomarkers for personalized treatment strategies [7]. However, the specific impact of these variants on luminal B breast cancer remains underexplored.

Nevertheless, the use of NACT in luminal B patients continues to be debated, particularly in early-stage disease, with some studies indicating that adjuvant chemotherapy may yield superior outcomes due to the inherent delays in surgery associated with NACT.

Given this backdrop, our study aims to investigate the influence of BRCA PVs on the likelihood of achieving pCR in luminal B breast cancer patients undergoing NACT. By comparing pCR rates and survival outcomes between patients with BRCA PVs and those with BRCA wild-type (BRCA WT), we seek to identify predictors that could enhance treatment personalization. Ultimately, this research endeavors to bridge the knowledge gap regarding the role of BRCA mutations in luminal B breast cancer, offering insights that could optimize therapeutic approaches and improve patient prognoses in this challenging subtype.

## 2. Materials and Methods

### 2.1. Patient Characteristics

This retrospective, observational, single-center study evaluated patients with clinical stage I-III luminal B breast cancer undergoing NACT followed by surgery at the Fondazione Policlinico Universitario Agostino Gemelli IRCCS in Rome. Patients were enrolled from 1 January 2014 to 30 June 2023 and were followed until December 2024. Participants were stratified into two groups based on BRCA mutation status: those with pathogenic variants and those who were BRCA wild-type.

Exclusion criteria included the following:

1. History of synchronous or previous systemic malignant cancer;
2. Previous ipsilateral or contralateral breast cancer;
3. Stage IV at diagnosis;
4. Evidence of metastatic spread during NACT;
5. Indication for hormonal neoadjuvant therapy.

Data were collected from patient records and entered into a database maintained by our Breast Unit.

### 2.2. Treatment Plan

A multidisciplinary team (MDT) consisting of breast and plastic surgeons, oncologists, radiation oncologists, radiologists, pathologists, geriatricians, psychologists, geneticists, and case managers tailored each patient’s treatment. Clinical staging (TNM classification) was determined through clinical evaluation, mammography, breast and axillary ultrasound, and magnetic resonance imaging (MRI). Prior to systemic treatment, a total body CT scan and bone scintigraphy were performed.

Clinical, pathological, and biological characteristics considered included the following: age at diagnosis, menopausal status (pre- or post-menopausal status at diagnosis), tumor location, size (assessed by MRI), multifocality/multicentricity (presence of lesions located in different quadrants or with multiple foci within the same breast), histotype, grading, expression of estrogen and/or progesterone receptors, proliferation index (Ki-67), and HER2 expression (categorized as HER2 0, HER2 1+, and HER2 2+ with SISH/FISH not amplified).

Genetic testing for BRCA 1 and BRCA 2 was conducted following international guidelines. Patients underwent germline genetic testing for BRCA1 and BRCA2 mutations using next-generation sequencing (NGS) technology. Detected variants in both genes were systematically analyzed and classified based on multiple reference databases, including ClinVar, the Leiden Open Variation Database (LOVD), and the Universal Mutation Database (UMD), as well as the relevant literature. Variants of uncertain significance (VUSs) were classified as BRCA wild-type, as they do not impact clinical decision-making in our routine practice.

Among the histological subtypes, the most common were ductal invasive carcinoma (DIC) and lobular invasive carcinoma (LIC). Other histological subtypes identified included invasive carcinoma of no special type (NST-IC), tubular invasive carcinoma (TIC), and medullary invasive carcinoma (MIC).

The predominant NACT regimen consisted of a sequential chemotherapy protocol: Epirubicin and Cyclophosphamide administered every three weeks, followed by weekly Paclitaxel for 12 cycles. The chemotherapy schedule was individualized for each patient by the MDT.

Following NACT, patients were re-staged with ultrasonography, mammography, and MRI to assess tumor response, categorized according to RECIST 1.1 criteria. Four types of radiological responses were identified: complete response (CR), partial response (PR), stable disease (SD), and progression of disease (PD). These imaging results informed the choice of surgical intervention which included quadrantectomy, level II oncoplastic breast surgery, conservative mastectomy, and radical mastectomy. Specifically, Level II oncoplastic procedures include volume-displacement techniques such as “inverted T mammoplasty”, “J mammoplasty”, “round block technique”, and “batwing mammoplasty”, which are used to reconstruct the defect resulting from the removal of 20–50% of the native breast tissue.

Axillary surgical approaches were determined based on clinical response. Patients with clinically negative lymph nodes (cN0-ycN0 or cN+-ycN0) underwent sentinel lymph node biopsy (SLNB) while those with macrometastasis in histological analyses underwent axillary dissection (AD). Patients with clinical or radiological evidence of lymph node involvement post-NACT (ycN+) also underwent direct axillary dissection.

pCR was defined as the absence of invasive breast cancer in both the breast and axillary lymph nodes. All patients received hormone therapy for up to 7–10 years, and radiation therapy was administered in accordance with applicable international guidelines.

Follow-up evaluations included semiannual outpatient visits for the first three years, transitioning to annual visits thereafter. Follow-up comprised locoregional assessments through breast and axillary ultrasound every six months and mammography annually. For patients with breast implants, annual MRI was also included. Systemic staging was conducted per established guidelines.

### 2.3. Endpoints

The primary aim of this study was to evaluate the rate of pCR between the two groups. Secondary endpoints included the assessment of locoregional disease-free survival (LR-DFS), defined as the time from surgery to locoregional recurrence; distant disease-free survival (DDFS), calculated from surgery to systemic relapse; and overall survival (OS), defined as the time from diagnosis or treatment initiation to death.

### 2.4. Statistical Analysis

Patients were stratified into two groups based on genetic testing results: BRCA1/2 with pathogenic variants (PV) and wild-type (WT). Continuous variables were described as mean ± standard deviation (SD) or median with interquartile range, and comparisons were made using Student’s t-test. Categorical variables were expressed as absolute numbers and percentages, with associations assessed using the chi-square test. Univariate and multivariable analyses were performed using binary logistic regression to identify factors associated with pCR, reporting odds ratios (ORs) with 95% confidence intervals. Survival curves were generated using the Kaplan–Meier method and compared using the log-rank test. All statistical analyses were two-tailed, with significance defined as *p* < 0.05. Analyses were conducted using SPSS version 26.0 (Statistical Package for Social Sciences).

## 3. Results

Between 1 January 2014 and 30 June 2023, a total of 1481 patients underwent neoadjuvant chemotherapy at Fondazione Policlinico Universitario Agostino Gemelli IRCCS. Of these, 590 patients (39.8%) had luminal B breast cancer while the remaining 891 patients (60.2%), who had triple-negative, HER2-enriched, or luminal A breast cancer, were excluded from the study. An additional 95 patients (6.4%) were excluded due to previous malignancy (31 patients, 2.1%), stage IV at diagnosis (24 patients, 1.6%), indication for neoadjuvant hormonal treatment (32 patients, 2.1%), or systemic spreading during chemotherapy (8 patients, 0.5%). Ultimately, 495 patients (33.4%) were included in the analysis.

**Figure 1:**
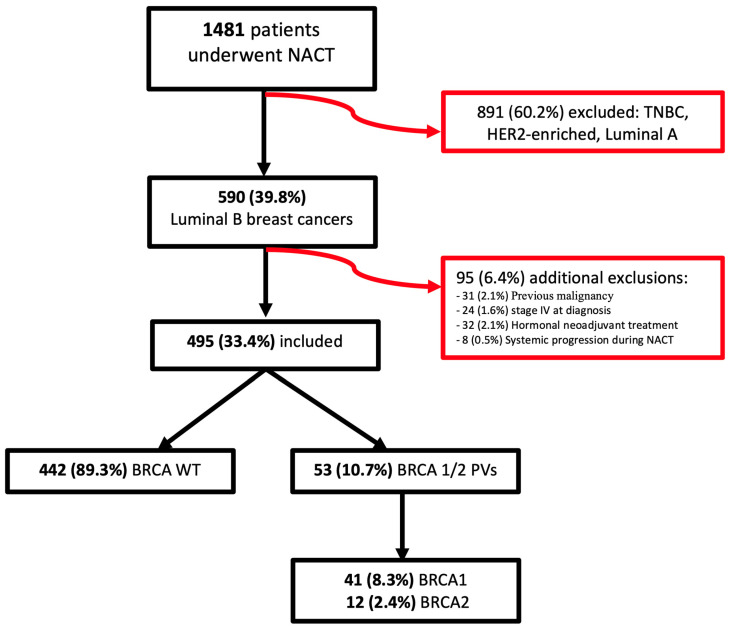
Flowchart of patients enrolled in this study.

Among the included patients, 53 (10.7%) had BRCA PVs: 41 patients (8.3%) had BRCA1 PVs, and 12 patients (2.4%) had BRCA2 PVs. The remaining 442 patients (89.3%) were BRCA WT (Figure 1).

### 3.1. Epidemiological Characteristics

Patients with BRCA1/2 PVs had a lower age of cancer onset (41.7 vs. 50.9 years; *p* < 0.0001) and lower menopausal status (18.9% vs. 51.1%; *p* < 0.0001) compared to WT patients. No significant differences were found in the biological characteristics of cancer (Table 1).

Table 1 shows the epidemiological and biological characteristics of the patients.

### 3.2. Radiological Assessment

For the initial radiological assessment, there were no significant differences between the two groups in cancer size at diagnosis (39.7 ± 20.1 mm vs. 40.1 ± 21.6 mm; *p* = 0.881), clinical T stage (*p* = 0.143), or N stage (*p* = 0.738). However, post-neoadjuvant assessments indicated that BRCA PV carriers had a higher number of complete responses (20 patients, (38.5%) vs. 106 patients (24.0%); *p* = 0.004) and a better overall response rate (48 patients (92.3%) vs. 308 patients (71.8%); *p* = 0.001). In addition, the size of residual cancer was smaller in BRCA PV carriers (19.1 ± 18.2 mm vs. 12.5 ± 17.6 mm; *p* = 0.014) (Table 2).

### 3.3. Surgical Treatment

BRCA PV carriers underwent conservative mastectomy with immediate reconstruction more frequently (40 patients (75.5%) vs. 124 patients (28.1%); *p* < 0.0001) and had fewer axillary radical treatments (27 patients (50.9%) vs. 302 patients (68.3%); *p* = 0.014) (Table 3).

Table 3 shows the type of surgical treatment on breast and axilla.

### 3.4. Pathological Evaluation

BRCA carriers exhibited a higher rate of pCR compared to WT patients (48 patients (20.8%) vs. 11 patients (10.9%); *p* = 0.044). ypT0 status was more prevalent among BRCA carriers (15 patients (28.3%) vs. 68 patients (15.4%); *p* = 0.005). Among BRCA carriers, the pCR rate was 9 out of 11 (81.8%) for BRCA1 and 2 out of 11 (18.2%) for BRCA2. After neoadjuvant chemotherapy, 12 (22.6%) of the 53 BRCA1/2 carriers converted to ypN0, compared to 55 (12.4%) of the 442 non-carriers (*p* = 0.054) (Table 4).

Table 4 shows the definitive histological evaluation.

### 3.5. Follow-Up Outcomes

**Figure 2:**
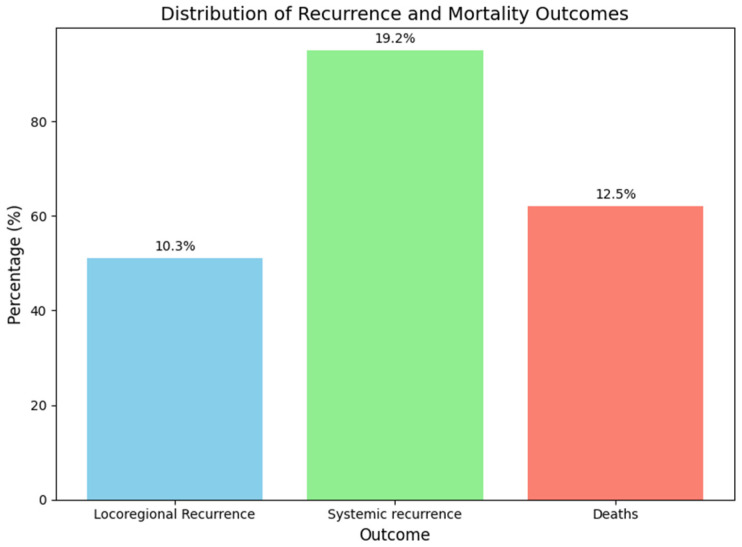
Distribution of recurrence and mortality.

After a median follow-up of 56.8 ± 73.9 months (58.3 months; range: 35.6–81.5 months), 51 patients (10.3%) reported locoregional recurrence: 20 in the axilla (4.0%), 25 in the breast (5.1%), and 6 in both (1.2%). Additionally, 95 patients (19.2%) reported systemic recurrence, and 62 patients (12.5%) died, primarily due to systemic disease progression. These outcomes were observed in the overall study population, not in a specific subgroup (Figure 2).

### 3.6. Oncological Outcomes

Patients with BRCA PVs showed an increased risk of locoregional recurrence despite a higher incidence of total mastectomy (12 patients (22.6%) vs. 39 patients (8.8%); *p* = 0.006). There were no differences regarding systemic recurrence or mortality rates (Table 5).

**Figure 3:**
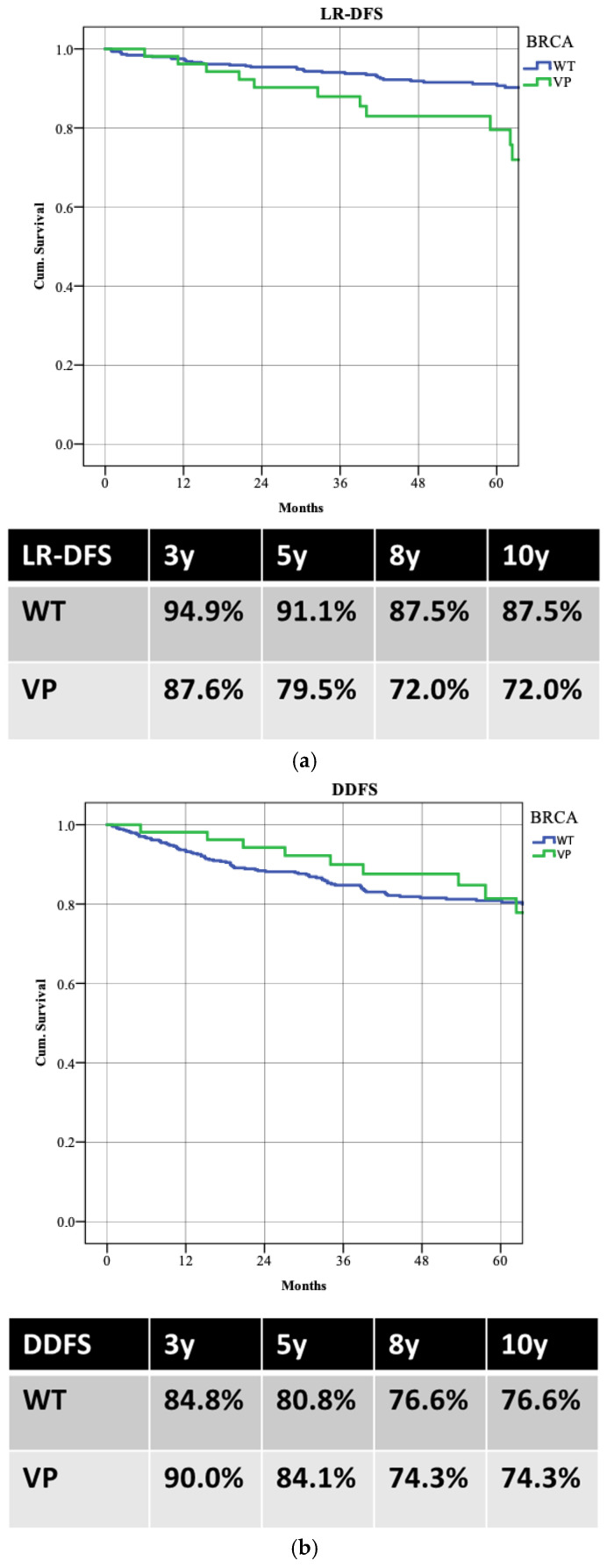

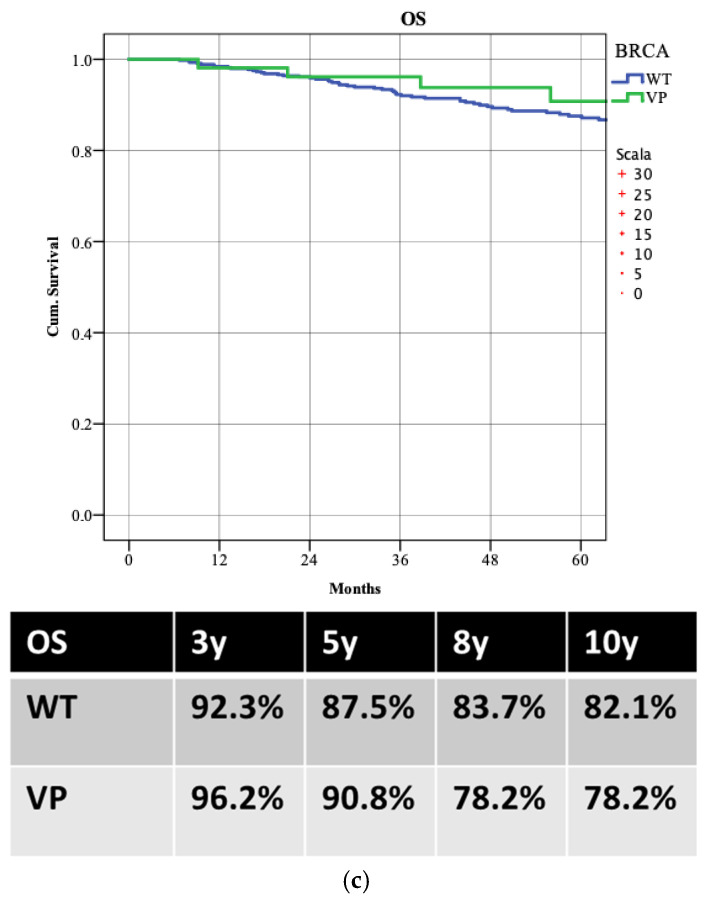
The survival rates between the two groups: (**a**) locoregional recurrence (LR-DFS); (**b**) distant recurrence (DDFS); (**c**) overall survival (OS).

**Figure 4:**
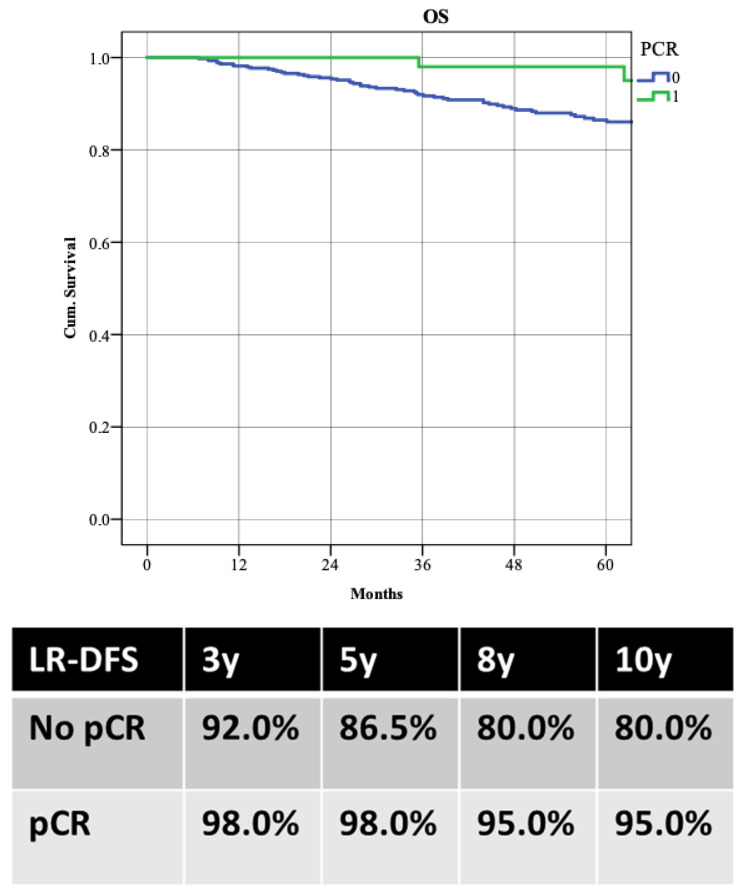
Overall survival (OS) according to the achievement of pathological complete response (pCR).

WT patients demonstrated better LR-DFS than those with PVs, with 5-year LR-DFS rates of 91.1% versus 79.5% (*p* = 0.003). Despite higher locoregional recurrence in PV patients, there was no significant difference in distant recurrence rates (5-year DDFS: 80.8% WT vs. 81.4% PVs; *p* = 0.853) or overall survival (5-year OS: 87.5% WT vs. 90.8% PVs; *p* = 0.988) (Figure 3). Patients achieving pCR had better survival rates, with a 5-year OS of 98.0% compared to 86.5% (*p* = 0.022) (Figure 4).

Table 5 describes the oncological outcomes of patients based on genetic status.

### 3.7. Univariate and Multivariable Analysis

Multivariable analysis identified several factors significantly associated with pCR: estrogen receptor status (*p* = 0.038), Ki-67 (*p* < 0.0001), and radiological response assessed by RECIST (*p* < 0.0001). Although the presence of BRCA PVs was significantly associated with pCR in the univariate analysis (*p* = 0.039), this association did not persist as an independent factor in the multivariable model, suggesting that the response to NACT is likely influenced by a combination of multiple biological and clinical factors. Other independent factors associated with pCR included age at onset (OR 0.969; *p* = 0.027), progesterone receptor status (OR 2.028; *p* = 0.036), clinical T stage (*p* = 0.002), and overall cancer stage (*p* = 0.001) (Table 6).

## 4. Discussion

NACT has significantly improved surgical and esthetic outcomes in patients with locally advanced breast cancer, facilitating the downstaging of the disease and enhancing options for breast and axillary conservative therapies [8]. Additionally, NACT provides valuable prognostic information based on breast and axillary response [9]. Assessing this response is crucial for tailoring adjuvant treatments and predicting recurrence risk [10]. Specifically, achieving pCR correlates with a reduced risk of disease relapse during follow-up while tumors showing progression during treatment demonstrate a markedly higher probability of recurrence even within short intervals [11]. Multiple studies have demonstrated that patients with HR-positive disease who achieve pCR also have improved survival outcomes relative to those who did not achieve complete response [12]. Pathologic complete response has predicted long-term outcomes in several neoadjuvant studies and is therefore a potential surrogate predictor of survival [13]. However, luminal B BC is less likely to respond to NACT than other biological subtypes. It is estimated that approximately 15% of patients with luminal B breast cancer achieve pCR after NACT [14,15]. Factors such as a high proliferation rate and the absence of progesterone receptors are positively associated with increased rates of pCR. Unfortunately, there are currently no fully reliable predictive factors, necessitating a complex, multidisciplinary approach to determine which patients will benefit most from NACT on a case-by-case basis [16]. Several studies suggest that regardless of tumor subtype, the response to treatments and prognosis varies for BRCA carrier patients [17]. This study provides significant insights into the relationship between BRCA PVs and pCR rates in luminal B breast cancer patients undergoing NACT. Our findings demonstrate that patients harboring BRCA PVs exhibit a markedly higher rate of pCR compared to their BRCA WT counterparts (20.8% vs. 10.9%; *p* = 0.044). This suggests that the presence of BRCA PVs could be an important biomarker for predicting treatment response in this specific patient population [18].

The higher pCR rates observed in BRCA PV carriers align with the established understanding that these variants compromise DNA repair mechanisms, making tumors more susceptible to the cytotoxic effects of chemotherapy [7]. The biological rationale lies in the fact that BRCA1 and BRCA2 genes are integral to the homologous recombination repair pathway [19]. Their dysfunction leads to genomic instability, which, in turn, can increase the efficacy of DNA-damaging agents used in chemotherapy regimens [20].

Several preclinical studies have demonstrated that BRCA1/2-related breast cancer cell lines exhibit varying degrees of sensitivity to chemotherapeutic agents that induce DNA damage, such as poly (ADP-ribose) polymerase (PARP) inhibitors [21].

Thus, the favorable response rates in BRCA PVs not only corroborate the existing literature but also highlight the need for genetic testing in luminal B breast cancer patients to inform treatment strategies [22]. These findings support the need for a broader consideration of BRCA genetic testing beyond age criteria alone. Genetic testing is not universally recommended for all early breast cancer patients [23]. Although traditional guidelines have prioritized BRCA testing in patients under 50 years of age, updated recommendations, including those from the NCCN, emphasize additional clinical indicators in the luminal B subtype such as nodal involvement (N2 stage), bilateral cancer, multicentric cancer, and a high CPS-EG score (≥3) as relevant criteria for testing [24]. Our study highlights that even within early-stage breast cancer, BRCA mutation status can meaningfully influence the response to neoadjuvant chemotherapy. This reinforces the potential utility of BRCA testing as part of a more personalized treatment strategy, particularly in selecting candidates for intensified treatment or PARP inhibitor therapy.

Interestingly, a recent study by the Memorial Sloan Kettering Cancer Center [7] highlighted a distinction between BRCA1 and BRCA2 mutations, finding a statistically significant association only between BRCA1 PVs and pCR, whereas BRCA2 PVs did not correlate with higher pCR rates. In our analysis, among BRCA carriers, the pCR rate was 81.8% for BRCA1 and only 18.2% for BRCA2. This disparity emphasizes the need for further investigation into the differential impact of these mutations on treatment outcomes.

**Figure 5:**
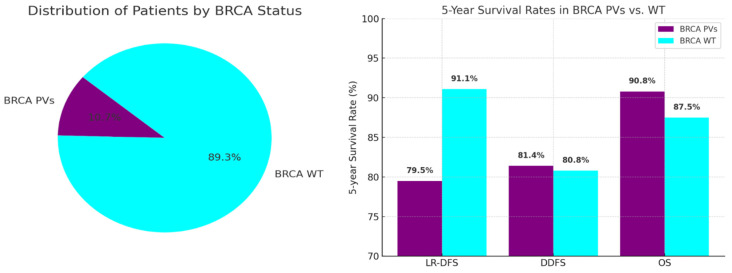
Oncological outcomes comparing BRCA WT and PV patients.

Moreover, our analysis indicates that BRCA WT patients, while experiencing lower pCR rates, demonstrate better LR-DFS at five years (91.1% vs. 79.5%; *p* = 0.003) (Figure 5). This raises important considerations regarding the long-term implications of treatment response [25]. Despite the apparent benefits of achieving pCR, the higher recurrence rates observed in BRCA PV carriers suggest that these patients may require more intensive follow-up and perhaps additional therapeutic interventions post-surgery [26]. The nuances of these outcomes underscore the complexity of managing luminal B breast cancer and the necessity for a tailored approach based on individual genetic profiles [27].

Moreover, while no significant differences were noted in DDFS and OS between the two groups, the trend towards increased locoregional recurrence in BRCA PV carriers necessitates further exploration into the underlying mechanisms. It could be that while BRCA PV patients respond better to initial chemotherapy, their tumors may possess intrinsic characteristics that predispose them to local recurrence, potentially due to factors unrelated to their response to NACT.

Another critical aspect of this study is the surgical treatment patterns observed in the two groups. Our data highlight that the breast pCR rate was 28.3% among BRCA PVs versus 15.4% among BRCA WT. Breast downstaging alone may have limited impact on the surgical management of BRCA PV carriers, as these patients may still be candidates for prophylactic mastectomy to reduce a second cancer event. Notably, BRCA PV carriers were more likely to undergo conservative mastectomy with immediate reconstruction (75.5% vs. 28.1%; *p* < 0.0001) and less likely to require axillary radical treatments (50.9% vs. 68.3%; *p* = 0.014). While breast-conserving surgery (BCS) may provide favorable cosmetic outcomes, it is essential to discuss the implications of surgical choices in BRCA mutation carriers [28]. Given their significantly elevated risk of developing second primary breast cancers, both contralateral and ipsilateral, many clinicians recommend mastectomy as a more proactive approach. This surgical option not only aims to eliminate the current tumor but also serves a prophylactic purpose, reducing the likelihood of future malignancies. Bilateral prophylactic mastectomy has been shown to reduce the incidence of breast cancer in patients with BRCA mutation, but several studies show no statistically significant improvement in survival among women undergoing contralateral risk-reduction mastectomy and intensive radiological surveillance [29].

When counseling patients, it is crucial to consider the benefits and drawbacks of both BCS and mastectomy with reconstruction. The choice of surgery should be tailored to the patient’s individual risk profile, preferences, and overall treatment goals. Engaging in a thorough discussion about these options empowers patients to make informed decisions that align with their values and concerns, particularly in light of their increased risk for second tumors [30].

On the other hand, achieving lymph node pCR may induce de-escalation in the surgical treatment of the axilla. After NACT, 12 (22.6%) of the 53 BRCA PV carriers converted to ypN0 compared with 55 (12.4%) of the 442 non-carriers (*p* = 0.054). Indeed, understanding the rate of axillary downstaging may significantly influence the choice of NACT versus upfront surgery. In fact, NACT may reduce the burden of axillary disease and provide an opportunity to avoid the morbidity of axillary lymph node dissection (ALND). Among the BRCA carriers, 83,3% (10/12) of the BRCA1 carriers in our study converted from node-positive to -negative compared with 16.7% (2/12) of the BRCA2 carriers. However, these differences were not statistically significant, likely due to the small sample size. The high lymph node pCR rate among BRCA1 carriers has demonstrated the efficacy of NACT in axillary downstaging and the de-escalation of surgical treatment and should be strongly considered.

Although the single-center design may limit the generalizability of the findings, our results are consistent with previous studies [15,31] that have demonstrated a higher frequency of global pathological complete response (pCR) or lymph node pCR in BRCA1 mutation carriers compared to BRCA2 carriers and non-carriers.

This study’s strengths lie in its large cohort size and the comprehensive analysis of treatment outcomes across multiple survival metrics. The use of a single-center design ensures consistency in treatment protocols and follow-up, enhancing the reliability of the findings. However, several limitations must be acknowledged. The findings of this study cannot be readily generalized due to its retrospective design, which may lead to potential type I and II statistical errors and selection bias from the heterogeneity of the study population. Moreover, the absence of a matched control group and the lack of randomization expose the analysis to further selection biases and confounding factors, potentially influencing the observed associations.

Additionally, while the study provides valuable insights, it does not delve deeply into the biological differences between BRCA1 and BRCA2 carriers, which could further elucidate their distinct responses to NACT. We acknowledge this limitation and recognize that future prospective, multi-center studies involving larger, independent cohorts are needed to validate our findings and to build more robust predictive models with better external applicability.

## 5. Conclusions

In conclusion, our findings suggest that BRCA pathogenic variants are significant predictors of pathologic complete response in luminal B breast cancer patients undergoing neoadjuvant chemotherapy. These results underscore the potential for integrating genetic testing into clinical practice, allowing for more personalized treatment strategies that could enhance patient outcomes. Future research should focus on understanding the biological mechanisms underlying the differences in treatment response and the long-term effects of BRCA PVs on recurrence patterns. This will be crucial in optimizing therapeutic approaches and improving prognoses for patients with this challenging breast cancer subtype.

## Figures and Tables

**Table 1:** Patient epidemiological and biological characteristics.

Characteristics	495 Patients	BRCA WT 442 (89.3%)	BRCA PV 53 (10.7%)	*p*-Value
Epidemiological Characteristics				
Age (years)	50 ± 10.6 (58.9; 42.3–56.2)	50.9 ± 10.3 (49.9; 43.8–57.5)	41.7 ± 9.5 (40.2; 34.7–46.5)	*p < 0.0001*
Menopausal status	236 (47.7%)	226 (51.1%)	10 (18.9%)	*p < 0.0001*
BMI (Kg/m^2^)	25.3 ± 5 (24.2; 22.0–27.3)	25.4 ± 5 (24.3; 22.2–27.4)	24.5 ± 4.7 (23.9; 21.1–26.9)	*p = 0.234*
Biological Characteristics				
Histotype				*p = 0.871*
- Ductal	316 (63.8%)	283 (64.0%)	33 (62.3%)	
- Lobular	39 (7.9%)	34 (7.7%)	5 (9.4%)	
- Other	140 (28.3%)	125 (28.3%)	15 (28.3%)	
Grading				*p = 0.371*
- G1	1 (0.2%)	1 (0.2%)	0 (0%)	
- G2	183 (37.0%)	167 (37.8%)	16 (30.2%)	
- G3	311 (62.8%)	274 (62.0%)	37 (69.8%)	
Estrogen Receptor				*p = 0.135*
- Negative	19 (3.8%)	15 (3.4%)	4 (7.5%)	
- Positive	476 (96.2%)	427 (96.6%)	49 (92.5%)	
Progesterone Receptor				*p = 0.839*
- Negative	72 (14.5%)	64 (14.5%)	8 (15.1%)	
- Positive	423 (85.5%)	378 (85.5%)	45 (84.9%)	
Androgen Receptor				*p = 0.837*
- Negative	71 (14.3%)	63 (14.3%)	8 (15.1%)	
- Positive	424 (85.7%)	379 (85.7%)	45 (84.9%)	
Ki67				*p = 0.160*
- <20%	4 (0.8%)	4 (0.9%)	0 (0%)	
- 20–50%	306 (61.8%)	279 (63.1%)	27 (50.9%)	
- >50%	185 (37.4%)	159 (36.0%)	26 (49.1%)	
HER2				*p = 0.698*
- 0	175 (36.0%)	154 (35.4%)	21 (41.2%)	
- 1+	166 (34.2%)	149 (34.3%)	17 (33.3%)	
- 2+	145 (29.8%)	132 (30.3%)	13 (25.5%)	

**Table 2:** Radiological baseline features and response to chemotherapy treatment.

Characteristics	495 Patients	BRCA WT 442 (89.3%)	BRCA PV 53 (10.7%)	*p*-Value
Baseline				
Cancer size (mm)	39.7 ± 20.2 (35; 25.0–50.0)	39.7 ± 20.1 (35; 26.0–50.0)	40.1 ± 21.6 (35; 25.0–50.0)	*p = 0.881*
Multifocality/Multicentricity	227 (45.9%)	205 (46.4%)	22 (41.5%)	*p = 0.561*
Clinical T stage				*p = 0.143*
- cT0	3 (0.6%)	3 (0.7%)	0 (0%)	
- cT1	52 (10.5%)	42 (9.5%)	10 (18.9%)	
- cT2	301 (60.8%)	273 (61.8%)	28 (52.8%)	
- cT3	86 (17.4%)	74 (16.7%)	12 (22.6%)	
- cT4	53 (10.7%)	50 (11.3%)	3 (5.7%)	
Clinical N stage				*p = 0.738*
- cN0	152 (30.7%)	134 (30.3%)	18 (34.0%)	
- cN1	229 (46.3%)	206 (46.6%)	23 (43.4%)	
- cN2	95 (19.2%)	86 (19.5%)	9 (17.0%)	
- cN3	19 (3.8%)	16 (3.6%)	3 (5.7%)	
Stage of cancer				*p = 0.064*
- 1A	14 (2.8%)	9 (2.0%)	5 (9.4%)	
- 1B	30 (6.1%)	26 (5.9%)	4 (7.5%)	
- 2A	98 (19.8%)	88 (19.9%)	10 (18.9%)	
- 2B	164 (33.1%)	150 (33.9%)	14 (26.4%)	
- 3A	118 (23.8%)	105 (23.8%)	13 (24.5%)	
- 3B	51 (10.3%)	48 (10.9%)	3 (5.7%)	
- 3C	20 (4.0%)	16 (3.6%)	4 (7.5%)	
Radiological Features Post-NACT				
RECIST criteria				*p = 0.003*
- Complete response	126 (26.2%)	106 (24.7%)	20 (38.5%)	
- Partial response	230 (47.8%)	202 (47.1%)	28 (53.8%)	
- Stable disease	114 (23.7%)	111 (25.9%)	3 (5.8%)	
- Progressive disease	11 (2.3%)	10 (2.3%)	1 (1.9%)	
Overall Response Rate	356 (74.0%)	308 (71.8%)	48 (92.3%)	*p = 0.001*
Residual cancer size (mm)	18.4 ± 18.3 (15; 0.03–46.0)	19.1 ± 18.2 (16; 3.1–28.0)	12.5 ± 17.6 (8.5; 0–17.3)	*p = 0.014*

**Table 3:** Surgical treatment of breast and axilla.

Characteristics	495 Patients	BRCA WT 442 (89.3%)	BRCA PV 53 (10.7%)	*p*-Value
Breast Surgery				
Type of Surgery:				*p < 0.0001*
- Lumpectomy/Quadrantectomy	208 (42.0%)	201 (45.5%)	7 (13.2%)	
- Level II Oncoplastic Surgery	71 (14.3%)	68 (15.4%)	3 (5.7%)	
- Conservative Mastectomy	164 (33.1%)	124 (28.1%)	40 (75.5%)	
- Radical Mastectomy	52 (10.5%)	49 (11.1%)	3 (5.7%)	
Axillary Surgery				
Sentinel Node Biopsy	385 (77.8%)	342 (77.4%)	43 (81.1%)	*p = 0.604*
Axillary Dissection	329 (66.5%)	302 (68.3%)	27 (50.9%)	*p = 0.014*

**Table 4:** Definitive histological characteristics of enrolled patients.

Characteristics	495 Patients	BRCA WT 442 (89.3%)	BRCA PV 53 (10.7%)	*p*-Value
Breast				
pCR	59 (11.9%)	48 (10.9%)	11 (20.8%)	*p = 0.044*
Breast pCR	83 (16.8%)	68 (15.4%)	15 (28.3%)	*p = 0.030*
Node pCR *	67 (13.5%)	55 (12.4%)	12 (22.6%)	*p = 0.054*
ypT				*p = 0.005*
- 0	83 (16.8%)	68 (15.4%)	15 (28.3%)	
- 1	279 (56.4%)	248 (56.1%)	31 (58.5%)	
- 2	106 (21.4%)	102 (23.1%)	4 (7.5%)	
- 3	21 (4.2%)	20 (4.5%)	1 (1.9%)	
- 4	6 (1.2%)	4 (0.9%)	2 (3.8%)	
Axillary				
Sentinel Node Biopsy				
- Removed lymph nodes	4.69 (5; 1–7)	4.70 (5; 1–7)	4.69 (5; 2–7)	*p = 0.827*
- Positive lymph nodes	0.71 (0; 0–6)	0.73 (0; 0–6)	0.61 (0; 0–5)	*p = 0.652*
Axillary Dissection				
- Removed lymph nodes	14.01 (13; 8–45)	14 (13; 8–45)	14.08 (13; 9–25)	*p = 0.943*
- Positive lymph nodes	3.39 (2; 0–33)	3.5 (2; 4.24–33)	2.08 (1; 0–16)	*p = 0.095*
ypN				*p = 0.061*
- 0	153 (30.9%)	131 (29.6%)	22 (41.5%)	
- 1	228 (46.1%)	202 (45.7%)	26 (49.1%)	
- 2	94 (19.0%)	90 (20.4%)	4 (7.5%)	
- 3	20 (4.0%)	19 (4.3%)	1 (1.9%)	

* rate considering patients with positive nodal disease.

**Table 5:** Oncological outcomes of enrolled patients.

Characteristics	495 Patients	BRCA WT 442 (89.3%)	BRCA PV 53 (10.7%)	*p*-Value
Locoregional recurrence				
Total	51 (10.3%)	39 (8.8%)	12 (22.6%)	*p = 0.006*
- Axilla	20 (4.0%)	17 (3.8%)	3 (5.7%)	
- Breast	25 (5.1%)	18 (4.1%)	7 (13.2%)	
- Both Axilla and Breast	6 (1.2%)	4 (0.9%)	2 (3.8%)	
Systemic recurrence				
	95 (19.2%)	85 (19.2%)	10 (18.9%)	*p = 1.000*
Exitus				
	62 (12.5%)	55 (12.4%)	7 (13.2%)	*p = 0.828*

**Table 6:** Univariate and multivariable analysis for predictive factors for pCR.

Characteristics	Univariate Analysis			Multivariable Analysis		
	OR	*p*-Value	95% CI	OR	*p*-Value	95% CI
Age (years)	0.969	0.027	0.943–0.996		0.735	
Menopausal Status						
- No	Ref.	Ref.	Ref.			
- Yes	0.725	0.253	0.417–1.259			
BRCA PV					0.738	
- No	Ref.	Ref.	Ref.			
- Yes	2.150	0.039	1.038–4.454			
Histotype		0.965				
- DIC	0.923	0.789	0.513–1.659			
- LIC	0.010	0.998	0.002–0.001			
- IC NST	Ref.	Ref.	Ref.			
Grading		0.102				
- 1	Ref.	Ref.	Ref.			
- 2	0.001	1.000	0.002–0.001			
- 3	0.001	1.000	0.002–0.001			
Estrogen Receptor						
- Negative	3.684	0.011	1.342–10.100	6.053	0.038	1.103–33.224
- Positive	Ref.			Ref.		
Progesterone Receptor						
- Negative	2.028	0.036	1.048–3.925		0.342	
- Positive	Ref.					
Androgen Receptor						
- Negative	1.077	0.855	0.488–2.377			
- Positive	Ref.					
Ki67	1.037	<0.0001	1.023–1.052	1.037	<0.0001	1.017–1.057
cT		0.002			0.096	
- 0	Ref.					
- 1	0.889	0.926	0.075–10.526			
- 2	0.315	0.351	0.028–3.557			
- 3	0.024	0.018	0.001–0.526			
- 4	0.001	0.997	0.001–0.002			
cN		0.120				
- 0	Ref.					
- 1	0.515	0.033	0.280–0.947			
- 2	0.570	0.158	0.261–1.243			
- 3	0.269	0.211	0.034–2.107			
Stage		0.001			0.618	
- 1A	Ref.					
- 1B	0.485	0.287	0.128–1.838			
- 2A	0.342	0.071	0.106–1.098			
- 2B	0.124	0.001	0.038–0.410			
- 3A	0.123	0.001	0.036–0.427			
- 3B	0.001	0.997	0.000 –			
- 3C	0.070	0.022	0.007–0.681			
Radiological Assessment (RECIST)		<0.0001			<0.0001	
- Complete Response	Ref.			Ref.		
- Partial Response	0.084	<0.0001	0.042–0.171	0.081	<0.0001	0.036–0.182
- Stable Disease	0.015	<0.0001	0.002–0.110	0.015	<0.0001	0.002–0.121
- Progressive Disease	0.000	0.999	0.000 –	0.000	0.999	0.000

## Data Availability

The data presented in this study are available on request from the corresponding author.

## Abbreviations

The following abbreviations are used in this manuscript

NACT	neoadjuvant chemotherapy
pCR	pathologic complete response
BRCA PVs	pathogenic variants in the BRCA gene
BRCA WT	BRCA wild-type gene
LR-DFS	locoregional disease-free survival
DDFS	distant disease-free survival
OS	overall survival
MDT	multidisciplinary team
